# Social Media Addiction and Orthorexia Nervosa Tendencies in Turkish Adults: A Cross-Sectional Study of BMI and Lifestyle Factors

**DOI:** 10.3390/bs16060864

**Published:** 2026-05-28

**Authors:** Bekir Erhan Orhan

**Affiliations:** Faculty of Sports Sciences, Istanbul Aydın University, Istanbul 34295, Türkiye; bekirerhanorhan@aydin.edu.tr

**Keywords:** social media, addiction, orthorexia nervosa, body mass index, lifestyle factors

## Abstract

This cross-sectional study examined whether addictive-like social media use, operationalized as generalized symptom-like engagement captured by the Bergen Social Media Addiction Scale (BSMAS) rather than content-specific exposure, is associated with orthorexia nervosa tendency in Turkish adults, and whether these associations persist after adjustment for BMI and lifestyle factors. Complete-case data on *n* = 258 adults (age ≥ 18 years) were analyzed using validated Turkish versions of the BSMAS and the Orthorexia Nervosa Inventory (ONI). Group differences were tested using Welch’s procedures with Holm-adjusted post hoc comparisons; associations were examined using Pearson correlations and HC3-robust multiple regression controlling for gender, age, education, physical activity frequency, smoking, alcohol use, and BMI. The mean age was 23.96 ± 9.36 years, and the mean BMI was 23.46 ± 4.09 kg·m^−2^. Men reported higher ONI total (g = 0.45) and higher BMI (g = 0.72) than women, whereas BSMAS did not differ by gender (g = 0.05). ONI total differed across BMI categories, with lower scores in the underweight category than in the overweight and obesity categories (Holm-adjusted *p* < 0.05). BMI showed a small positive correlation with ONI total (r = 0.136), and age was weakly negatively correlated with BSMAS (r = −0.172). BSMAS showed small correlations with ONI Impairments and ONI Emotions (r = 0.139–0.148). The bivariate association between BSMAS and ONI total was small and non-significant (r = 0.092). BSMAS was not an independent predictor of ONI total in the covariate-adjusted HC3 regression (B = 0.246, *p* = 0.141; adjusted R^2^ = 0.059). In this sample of Turkish adults, orthorexia nervosa tendency was associated with gender and BMI strata. In contrast, generalized addictive-like social media use showed no independent association with overall orthorexia after covariate adjustment. The absence of an independent BSMAS-ONI association should not be interpreted as evidence that social media is unrelated to orthorexia; rather, a generalized addiction-like measure may be insufficient to capture content- and platform-specific digital pathways. Future research should incorporate content-specific exposure and longitudinal designs to clarify mechanisms.

## 1. Introduction

Social media has become a dominant platform through which health, fitness, and “clean eating” norms are created, circulated, and normalized, making dietary ideals highly visible and socially negotiable (Goodyear et al., 2021). These platforms expose users to nutrition tips, transformation narratives, and appearance-focused imagery that may support behavior change while simultaneously intensifying body surveillance, comparison, and rule-driven conceptions of health (Wu et al., 2024; Zaharia & Gonţa, 2024). Because engagement is heterogeneous, a subset of users reports excessive and difficult-to-regulate use patterns that resemble behavioral addiction and are associated with psychosocial costs when online activity displaces sleep, academic/work routines, and in-person relationships (Shannon et al., 2022; Weinstein, 2023; Kolhar et al., 2021; Zhuang et al., 2023). Social media can therefore operate as both an informational resource for exercise and nutrition and a context that magnifies exposure to idealized bodies and sedentary screen time (Orhan et al., 2025).

A conceptual distinction is essential for interpreting empirical work in this area. Generalized addictive-like social media use, characterized by preoccupation, tolerance, withdrawal-like states, conflict, and unsuccessful regulation attempts across platforms, is conceptually and operationally distinct from content-specific exposure to platforms or feeds dominated by fitness, “clean eating,” body-transformation, or influencer-driven nutrition content (Athanasoula et al., 2025; Sharma & Vidal, 2023; Usta Ulutaş & Okan Bakır, 2025). Eating-related risk may depend less on total time online than on platform-specific content environments and the ways users interact with them. Most validated instruments, including the Bergen Social Media Addiction Scale (BSMAS), capture the generalized dimension; they do not index exposure to fitspiration, clean-eating communities, or body-comparison content. This conceptual measurement gap is central to the interpretation of the present findings and is revisited throughout. 

The tendency toward orthorexia nervosa is highly relevant within this digital ecology because contemporary wellness discourse frequently frames dietary purity as a moral virtue and personal identity, creating fertile ground for rigid eating rules (Usta Ulutaş & Okan Bakır, 2025; Gonçalves, 2025). Orthorexia-related patterns are typically conceptualized as maladaptive preoccupation with “healthy” eating that involves inflexibility, distress when rules are violated, and, at more severe levels, functional impairment or nutritional compromise (Horovitz & Argyrides, 2023; Ng et al., 2024). In contrast to adaptive health motivation, orthorexia tendencies often include self-worth contingencies, compulsive monitoring, and escalating restrictions that can backfire by reducing dietary flexibility and social functioning (Gortat et al., 2021; Mahfoud et al., 2023). Although orthorexia is not a formal diagnostic category, advances in measurement have strengthened research assessment and cross-cultural comparability, including multidimensional instruments that capture behavioral, emotional, and impairment components (Cerolini et al., 2022; Zagaria et al., 2023).

Several mechanisms have been proposed to connect addictive-like social media use with orthorexia tendencies, although direct empirical evidence remains limited and is largely cross-sectional. Algorithmically curated feeds can repeatedly foreground diet- and body-related ideals, potentially reinforcing social comparison and perceived norms about “optimal” eating (Vandenbosch et al., 2022; Wu et al., 2024). Influencer-driven messaging may further blur epistemic trust cues, making anecdotal claims appear equivalent to professional guidance and co-occurring with rule-based dietary practices (Friedman et al., 2022; Dessì et al., 2025). Engagement-driven recommender systems can also create “content funnels,” in which interaction with fitness or nutrition posts is associated with progressively more extreme dietary content, which may co-occur with preoccupation and reduced flexibility (Oh & Lee, 2025; Zeng et al., 2025). Content analyses describe active orthorexia-oriented online communities that moralize dietary purity and frame restrictive practices as identity-relevant (Zemlyanskaya et al., 2022; Valente et al., 2022). Consistent with these pathways, survey studies have reported associations between social media addiction symptoms, interaction with nutrition content, and elevated orthorexia-related outcomes (Yurtdaş-Depboylu et al., 2022; Vintró-Alcaraz et al., 2026; Gul & Koc, 2025).

At the same time, the social media addiction–orthorexia association may be confounded by body mass index (BMI) and broader lifestyle characteristics. BMI correlates with weight concern, exposure to stigma, and engagement in weight-control practices, all of which may be associated with both diet-related preoccupation and online health information seeking (K. M. Lee et al., 2021; Tan et al., 2025). Individuals with higher BMI may experience stronger pressures to manage weight and may perceive “clean eating” narratives as especially salient, potentially elevating orthorexia-related scores independent of generalized addictive-like use (Davies et al., 2022; dos Santos Avelar & Laus, 2025). Lifestyle factors, such as physical activity frequency, smoking, alcohol use, and education, may further be associated with health-related attitudes and self-regulatory patterns and thus need to be considered when evaluating digital exposure hypotheses (Ardesch et al., 2023; Gheonea et al., 2023; Orhan et al., 2026a, 2026b).

Empirical evidence from Türkiye (Turkey) is particularly relevant given high internet penetration and widespread use of social networks and online health-information seeking among youth and adults, which may interact with local food cultures and globalized wellness ideals (Turkish Statistical Institute, 2020, 2024; Ammar et al., 2025; Pehlivan et al., 2025). However, studies integrating validated measures of both social media addiction symptoms and multidimensional orthorexia symptomatology while accounting for BMI and lifestyle covariates remain limited.

Building on this background, the present study examined whether generalized addictive-like social media use, indexed by the BSMAS, remains associated with multidimensional orthorexia tendency, indexed by the ONI, after adjustment for BMI and lifestyle covariates in a Turkish adult sample. The study’s contribution is intentionally framed as modest and incremental: although associations between social media use and disordered eating outcomes have been examined in earlier Turkish and international samples, comparatively few studies have combined the multidimensional ONI with HC3-robust covariate-adjusted modeling in this population. It was hypothesized that higher BSMAS scores would be positively associated with ONI tendency, and that this bivariate association would attenuate after adjustment for BMI and lifestyle covariates, consistent with the possibility that weight-related concerns and content-specific exposure, rather than generalized addiction-like use, are the more proximal correlates of orthorexia-related symptoms. 

## 2. Materials and Methods

### 2.1. Study Design and Participants

This cross-sectional study used an anonymous online questionnaire administered in Türkiye. Data were collected between May 2025 and October 2025. Adults (≥18 years) who provided electronic informed consent were eligible. Recruitment used online convenience sampling through multiple digital channels. The survey link, hosted on Google Forms, was distributed via open calls on the principal investigator’s institutional networks at Istanbul Aydın University (faculty mailing lists, departmental announcements, and student WhatsApp groups), public posts on Instagram, X (formerly Twitter), and Facebook, and informal snowball forwarding by initial respondents. Because recruitment relied entirely on digital channels, the resulting sample is likely to over-represent individuals with above-average digital engagement, which is acknowledged as a selection-bias concern in the Limitations section. Participation was voluntary and uncompensated.

Of 312 individuals who opened the survey link and proceeded past the consent screen, 312 (100%) provided electronic informed consent and began the questionnaire. A total of 54 cases (17.3%) were excluded listwise because of missing values on at least one variable required for the analyses; no participant withdrew explicitly, and no respondent was excluded for failing age eligibility (all respondents were ≥18 years). The final analytic sample comprised complete-case data on *n* = 258 participants. Missingness was distributed across items rather than concentrated on a single variable; included and excluded participants did not differ on age (Welch t = 0.42, *p* = 0.674) or gender distribution (χ^2^ = 1.08, *p* = 0.30), reducing but not eliminating concerns about systematic attrition. Because the study collected no directly identifying information, individual-level non-response cannot be characterized further. The proportion excluded (17.3%) is non-trivial and is treated as a limitation. 

Several quality-control limitations of the online delivery should be acknowledged transparently. No technical procedures were implemented to prevent duplicate participation (no IP-based de-duplication, browser cookies, unique single-use links, or device identifiers). Attention-check items, minimum-completion-time thresholds, and straight-line response detection were not embedded in the instrument. Survey delivery was self-administered online without researcher supervision; participants completed all measures in a single session of approximately 8–12 min. These omissions are acknowledged as limitations and are returned to in Section Limitations and Future Directions. Reporting follows the relevant items of the STROBE checklist for observational studies and CHERRIES checklist for online surveys (von Elm et al., 2007; Eysenbach, 2004); core items are addressed throughout Section 2.1, Section 2.2, Section 2.3 and Section 2.4.

Participants completed validated Turkish versions of the BSMAS and ONI, along with items on sociodemographics and lifestyle behaviors (physical activity frequency, smoking, alcohol use) and self-reported anthropometrics. No directly identifying personal information was collected, and participants could discontinue at any point without penalty. The study protocol was reviewed and approved by the Social and Humanities Sciences Ethics Commission of Istanbul Aydın University (Meeting No. 2025/5). The study was conducted in accordance with the Declaration of Helsinki.

### 2.2. Measures

#### 2.2.1. Bergen Social Media Addiction Scale (BSMAS)

Generalized addictive-like social media use was assessed with the 6-item BSMAS. The BSMAS does not measure smartphone use, general internet use, or content-specific exposure to particular platforms or topics; rather, it captures the six core components of behavioral addiction adapted to generalized social media engagement salience (preoccupation), tolerance, mood modification, relapse (failure to cut down), withdrawal-like states, and conflict with each item indexing one component (Andreassen et al., 2016; Demirci, 2019). Items are rated on a 5-point Likert scale (1 = very rarely to 5 = very often) for the past 12 months and summed to yield a total score ranging from 6 to 30, with higher scores indicating greater addictive-like social media use (Demirci, 2019). In the Turkish adaptation, the one-factor structure showed acceptable fit in university students (χ^2^ = 10.80, df = 9, *p* = 0.290; CFI = 0.99; TLI = 0.99; SRMR = 0.031; RMSEA = 0.039), and was also supported in high-school students (χ^2^ = 16.02, df = 9, *p* = 0.066; CFI = 0.95; TLI = 0.92; SRMR = 0.048; RMSEA = 0.079) and working adults (χ^2^ = 11.98, df = 9, *p* = 0.214; CFI = 0.99; TLI = 0.98; SRMR = 0.039; RMSEA = 0.046) (Demirci, 2019). In the present sample, internal consistency was high (Cronbach’s α = 0.872). Throughout the manuscript, scores on the BSMAS are described as indexing addictive-like or symptom-like social media use rather than a confirmed clinical diagnosis. 

#### 2.2.2. Orthorexia Nervosa Inventory (ONI)

Orthorexia-related symptoms were measured using the 24-item ONI, which yields a total score (24–96) and three subscale scores: Behaviors (9–36), Impairments (10–40), and Emotions (5–20), with higher scores indicating greater orthorexic tendencies (Oberle et al., 2021). In the Turkish validation study, model fit indices supported acceptable fit (CMIN/df = 5.65; RMSEA = 0.08; CFI = 0.94; NFI = 0.93; SRMR = 0.07; IFI = 0.94) (Kaya et al., 2022). In the present sample, internal consistency was excellent for the total score (Cronbach’s α = 0.946) and good for the subscales (Behaviors α = 0.880; Impairments α = 0.909; Emotions α = 0.833). Consistent with the developers’ framing, ONI scores are interpreted as indicators of orthorexia-related tendency rather than a confirmed clinical diagnosis. 

### 2.3. Anthropometrics, Sociodemographic, and Lifestyle Covariates

Participants self-reported weight (in kilograms) and height (in meters). BMI (kg·m^−2^) was computed from these values and classified using WHO categories: underweight (<18.5), normal weight (18.5–24.9), overweight (25.0–29.9), and obesity (≥30.0) (World Health Organization Regional Office for Europe, 2025). Age was recorded continuously in years. Gender was assessed using a single binary item (women/men); this binary format is acknowledged as a methodological limitation in Section Limitations and Future Directions. Education was recorded in four ordered categories (Primary/lower, High school, University, Postgraduate). Physical activity frequency was assessed by a single self-report item: “How many days per week do you engage in physical activity?” with response options None, 1–2, 3–4, 5–6 days/week, or Daily; this item does not capture intensity, modality, or duration. Smoking status used three categories (Never, Former, Current). Alcohol use was recorded as None, Occasional, or Weekly+. 

### 2.4. Statistical Analysis

Variables were summarized as mean ± SD or *n* (%), and internal consistency was evaluated with Cronbach’s α. Group differences were tested with Welch’s *t*-tests and Welch’s ANOVA (Kruskal–Wallis as a sensitivity check), followed by Holm-adjusted pairwise Welch tests after significant omnibus results. Associations were assessed using Pearson correlations (r; 95% CIs) and OLS regression with HC3 robust standard errors, with model-specific reference categories. Effect sizes (Hedges g; ε^2^ for Kruskal–Wallis) were reported, with negative ε^2^ set to 0. ONI total was the primary outcome; subscale analyses were exploratory. Statistical significance was set at *p* < 0.05 (two-tailed). Regression diagnostics included: multicollinearity, evaluated with variance inflation factors (all VIFs < 2.5, well below the conventional threshold of 5); residual distribution, inspected through standardized-residual histograms and Q-Q plots, which did not indicate departures from normality of sufficient magnitude to invalidate inference; heteroscedasticity, addressed by the use of HC3-robust standard errors throughout; and influential observations, screened via Cook’s distance and standardized dfBETAs, with no case exceeding conventional thresholds (Cook’s D > 4/*n* or |dfBETA| > 1). The use of HC3 does not eliminate the need for these checks, which are reported here for transparency. Sensitivity power analyses in G*Power 3.1 (α = 0.05; power = 0.80) indicated that with *n* = 258 and 15 predictors, the minimum detectable effect was f^2^ = 0.077 (≈R^2^ = 0.071), and the minimum detectable correlation was |r| = 0.173 (Faul et al., 2009). Reporting follows the STROBE checklist for cross-sectional studies and relevant CHERRIES items for online surveys (von Elm et al., 2007; Eysenbach, 2004). 

## 3. Results

Across outcomes, robust group comparisons indicated significant differences in orthorexia nervosa tendency and BMI by gender, and significant differences in ONI total across BMI categories. In contrast, addictive-like social media use (BSMAS) did not differ by gender or BMI category. Omnibus tests further showed significant differences in BMI by education level. Because BMI categories are themselves derived from BMI, BMI-by-BMI comparisons are tautological and are not reported.

Men reported higher ONI total than women (51.03 ± 15.23 vs. 44.91 ± 11.68) and higher BMI (24.97 ± 3.78 vs. 22.17 ± 3.92). Following Cohen’s (1988) conventions, these effects can be interpreted as small to moderate for ONI total (g = 0.45) and moderate to large for BMI (g = 0.72). Post hoc analyses indicated that the ONI total was lower in the underweight category compared with the overweight and obesity categories (Holm-adjusted *p* < 0.05), with moderate-to-large effects (g = −0.65 to −0.99). Education-related post hoc results showed lower BMI in the Primary/lower and High school groups compared with the University group, and lower BMI in Primary/lower compared with Postgraduate; the University and Postgraduate groups did not differ significantly after Holm correction.

Correlation analyses showed a small positive association between BMI and ONI total (r = 0.136, *p* = 0.029) and a small negative association between age and BSMAS (r = −0.172, *p* = 0.006). In line with Cohen’s (1988) conventions, these correlations are best characterized as small in magnitude. In covariate-adjusted regression models with HC3 robust standard errors, BSMAS total was not an independent predictor of ONI total (B = 0.246, *p* = 0.141) after controlling for BMI, gender, age, education, physical activity frequency, smoking, and alcohol use. This null finding should not be interpreted as evidence that social media is unrelated to orthorexia tendency; rather, it indicates that the study did not find evidence of an independent association between generalized BSMAS scores and ONI total in this sample after adjustment for the selected covariates. 

### 3.1. Participant Characteristics

Table 1 presents the characteristics of the sample (*n* = 258). Mean age was 23.96 ± 9.36 years, and mean BMI was 23.46 ± 4.09 kg·m^−2^ (height: 1.71 ± 0.11 m; weight: 69.20 ± 16.60 kg). Average scores were 18.03 ± 6.20 for BSMAS and 47.74 ± 13.75 for ONI total (Behaviors: 19.06 ± 5.36; Impairments: 18.20 ± 6.28; Emotions: 10.48 ± 3.58). Women comprised 53.9% (men 46.1%). Education was mainly high school (64.3%), followed by university (22.9%), primary/lower (10.5%), and postgraduate (2.3%). Physical activity was most commonly 1–2 days/week (31.0%) or 3–4 days/week (29.1%); 12.0% reported none. Smoking status was never (57.0%), current (30.6%), and former (12.4%). Alcohol use was none (68.2%), occasional (25.6%), and weekly+ (6.2%). BMI categories were normal weight (60.9%), overweight (23.6%), underweight (9.3%), and obesity (6.2%).

Table 2 indicates good-to-excellent reliability. Cronbach’s α was 0.872 for BSMAS and 0.946 for ONI total; ONI subscales were also reliable (0.880–0.909), with all α values above 0.70.

Table 3 shows no gender difference in BSMAS (*p* = 0.675; g = 0.052). In contrast, men scored higher than women on ONI total and all ONI subscales (all *p* < 0.001; Emotions *p* = 0.038), with small-to-moderate effect sizes (g = 0.261–0.470 per Cohen, 1988). Men also had higher BMI than women (*p* < 0.001) with a moderate-to-large effect (g = 0.723).

Table 4 shows significant omnibus effects for gender on ONI total and BMI (both *p* < 0.001), but not on BSMAS (*p* = 0.675). BMI category was associated with ONI total (*p* = 0.005), but not with BSMAS (*p* = 0.948). Education differed for BMI only (*p* < 0.001); physical activity, smoking, and alcohol use showed no significant omnibus differences for BSMAS, ONI total, or BMI (all *p* > 0.05). As noted in the table caption, BMI-by-BMI comparisons are not reported because BMI categories are themselves derived from BMI.

Table 5 shows a graded increase in ONI total across BMI categories: Underweight had the lowest mean (42.17 ± 12.39), followed by Normal weight (46.58 ± 13.51), Overweight (51.20 ± 14.30), and Obesity, which had the highest mean (54.25 ± 11.32). Medians showed a similar pattern (42.0 to 55.5).

Table 6 indicates that BMI increased with higher education level: Primary/lower had the lowest mean BMI (21.75 ± 5.00), followed by High school (23.09 ± 3.77), University (24.93 ± 4.12), and Postgraduate, showing the highest mean (26.99 ± 2.35). Medians followed the same trend (19.7 to 26.8).

Table 7 shows that for the ONI total, only comparisons involving underweight remained significant after Holm correction: Underweight < Overweight (*p* = 0.029; g = −0.648, moderate effect) and Underweight < Obesity (*p* = 0.019; g = −0.988, large effect). Differences between normal weight vs. overweight/obesity were not significant after adjustment (*p* = 0.080–0.096). For education and BMI, BMI was lower in Primary/lower than University (*p* = 0.024) and Postgraduate (*p* = 0.008), and lower in High school than University (*p* = 0.016) and Postgraduate (*p* = 0.024).

Table 8 indicates uniformly small correlations following Cohen’s (1988) conventions. BSMAS was not correlated with ONI total (r = 0.092, *p* = 0.143) or ONI Behaviors (r = −0.027, *p* = 0.661) but showed small positive correlations with ONI Impairments (r = 0.139, *p* = 0.025) and ONI Emotions (r = 0.148, *p* = 0.017). BMI was weakly positively correlated with ONI total (r = 0.136, *p* = 0.029). Age was negatively correlated with BSMAS (r = −0.172, *p* = 0.006) and moderately positively correlated with BMI (r = 0.392, *p* < 0.001), while age was not associated with ONI total (r = 0.022, *p* = 0.725). Given multiple bivariate tests, the small subscale correlations should be interpreted with caution. 

### 3.2. Multivariable Models

Reference categories: Gender = Women; Physical activity = None; Smoking = Never; Alcohol use = None; Education = High school. All VIFs were below 2.5, indicating no collinearity concern; Cook’s distance and dfBETA inspections did not identify influential cases. 

Table 9 indicates that men had higher ONI total than women (B = 7.142, *p* = 0.001), while occasional alcohol use was associated with lower ONI total (B = −4.958, *p* = 0.027). BSMAS, BMI, age, and the other covariates were not significant (*p* > 0.05). Model fit was modest (R^2^ = 0.114; adjusted R^2^ = 0.059). Consistent with the interpretive caution adopted throughout this study, the non-significant BSMAS coefficient should not be interpreted as evidence of no relationship; rather, the analysis did not detect evidence of an independent association between generalized BSMAS scores and ONI total in this sample after adjustment for the included covariates. 

Table 10 shows that age was the only significant predictor of BSMAS (B = −0.173, *p* = 0.004); older participants reported lower BSMAS scores. All other predictors were non-significant (*p* > 0.05). Model fit was modest (R^2^ = 0.079; adjusted R^2^ = 0.025).

Table 11 indicates that men had higher BMI than women (B = 2.141, *p* < 0.001) and BMI increased with age (B = 0.168, *p* < 0.001). Physical activity, smoking, alcohol use, and education were not significant predictors (*p* > 0.05). Model fit was moderate (R^2^ = 0.246; adjusted R^2^ = 0.206).

## 4. Discussion

The present study evaluated whether addictive-like social media use is associated with orthorexia nervosa tendency in Turkish adults when BMI and lifestyle indicators are considered. Three results were most salient. Orthorexia-related outcomes and BMI differed by gender, whereas addictive-like social media use did not. Orthorexia tendency varied across BMI categories and showed a small positive bivariate association with BMI. Finally, the study did not find evidence that generalized addictive-like social media use is independently associated with overall orthorexia in covariate-adjusted models. This null finding is consistent with the possibility that a generalized BSMAS-based index does not capture the digital pathways most relevant to orthorexia-related symptomatology. The pattern, however, is not on its own sufficient to establish that interpretation and is offered as a hypothesis for future research. 

The present findings extend the existing Turkish and international literature on social media use and disordered eating in a specific and modest way. Earlier work has often relied on bivariate or partially adjusted analyses or on single-construct orthorexia measures with limited coverage of the four consensus diagnostic criteria. By combining the multidimensional ONI (which separately indexes behaviors, impairments, and emotions) with HC3-robust regression adjustment for BMI, education, physical activity, smoking, alcohol use, age, and gender, the study offers a methodologically more demanding test of whether generalized BSMAS-based addiction-like use independently tracks overall orthorexia tendency in a Turkish adult sample. The results show small bivariate correlations with the Impairments and Emotions subscales only, and no independent association with ONI total after adjustment, supporting the broader argument that content-specific exposure (e.g., fitspiration, clean-eating communities, influencer nutrition content) is plausibly the more proximal correlate of orthorexia-related symptoms than generalized addictive-like engagement. This argument should, however, be treated as an explanatory hypothesis for future research, because content-specific exposure was not directly measured in the present design. 

Gender differences in ONI scores, coupled with higher BMI among men, suggest that orthorexia-related concerns are associated with both men and women and may reflect gendered routes into dietary rigidity. The observed gender differences may be interpreted, cautiously and as a hypothesis for future research, in light of contemporary fitness cultures in which men may experience pressures related to physique optimization, performance nutrition, and strict dietary control. Such pressures, particularly within training-oriented communities, may be associated with rule-governed eating and distress when standards are unmet (Hilkens et al., 2021; Timm et al., 2025; Louw et al., 2025). Men may also be more likely to endorse impairment-related items captured by the ONI due to differences in routines, role expectations, or the salience of performance-linked eating practices (Hallit et al., 2021; Zagaria et al., 2023; Łucka et al., 2024). These interpretations are speculative because the present study did not measure gym involvement, muscularity-oriented concerns, supplement use, bodybuilding practices, training modality, or exposure to male-oriented fitness content. They should be tested directly in future research. The absence of gender differences in BSMAS scores is consistent with the possibility that symptom-like patterns are broadly distributed across genders, while gendered differences may emerge more clearly in platform preferences and content-specific engagement that the BSMAS does not capture (Brailovskaia & Margraf, 2024; Zarate et al., 2022). Gender in the present study was measured using a binary item (women/men); this is acknowledged in the Limitations as a methodological constraint. 

ONI total differed across BMI categories, with lower scores in the underweight group than in the overweight and obesity groups, and BMI showed a small positive correlation with orthorexia tendency. Higher BMI is often associated with stronger weight-control pressures and repeated dieting efforts, which may co-occur with preoccupation with “healthy” eating and facilitate rigid dietary rules (Chu et al., 2021; H. Y. Lee & Hong, 2021; Menniti et al., 2025). However, BMI was derived from self-reported anthropometric data, and potential reporting biases may attenuate or distort category assignments, underscoring the need for objective measurements when feasible.

The negative association between age and BSMAS scores indexing addictive-like social media use aligns with cohort differences in the centrality of social platforms for identity signaling, social feedback, and everyday coordination, which may co-occur with compulsive engagement among younger adults (Brailovskaia & Margraf, 2024; Casale et al., 2023). Yet orthorexia outcomes were not associated with age in this sample, suggesting that age-linked variation in symptom-like social media use does not straightforwardly translate into orthorexia-related symptom differences. This divergence supports arguments that the orthorexia risk may relate more strongly to platform type, content focus, and engagement patterns than to the general intensity of use (Athanasoula et al., 2025; Usta Ulutaş & Okan Bakır, 2025; Vintró-Alcaraz et al., 2026).

A primary aim was to test whether BSMAS predicts orthorexia after adjusting for BMI, age, and lifestyle covariates. The null association for ONI total in HC3-robust regression suggests that generalized addictive-like social media use does not uniquely track overall orthorexia tendency once weight status and lifestyle indicators are considered. This pattern is consistent with content- and community-specific pathways in which moralized nutrition discourse and “clean eating” environments may be more relevant than general compulsive use, even when bivariate associations are observed (Gonçalves, 2025; Valente et al., 2022; Usta Ulutaş & Okan Bakır, 2025). These content-specific mechanisms were not directly tested in the present study and should be regarded as explanatory hypotheses for future research rather than findings of the present work. The small correlations with ONI Impairments and Emotions, but not Behaviors or total, further suggest that if BSMAS-indexed addictive-like social media use relates to orthorexia in this population, the link may be most apparent in distress and functional interference rather than in the behavioral enactment of dietary rules (Vintró-Alcaraz et al., 2026).

Adjusting for lifestyle covariates strengthens interpretability by situating orthorexia-related symptoms within broader behavior profiles. Physical activity and education can index health literacy, motives, and exposure to fitness cultures, whereas smoking and alcohol use may reflect wider self-regulatory patterns that co-occur with dietary rigidity (Rozmiarek et al., 2024; Orhan et al., 2026b; Łucka et al., 2025). Nonetheless, the modest model fit and reliance on categorical covariate strata indicate that more granular measures, such as exercise type and intensity, dietary restraint, sleep disruption, body dissatisfaction, and explicit indicators of exposure to nutrition/fitness content, are likely needed to clarify mechanisms.

### Limitations and Future Directions

Several limitations should be noted. First, the cross-sectional design precludes causal inference and cannot establish temporal ordering between social media use and orthorexia-related symptoms. Second, anthropometric data and gender were self-reported; weight and height are subject to reporting biases that may distort BMI category assignment, and gender was assessed using a binary item, which does not capture the full diversity of gender identity and expression. Third, the sample was relatively young and was recruited entirely through digital channels (Instagram, X, Facebook, WhatsApp, institutional networks, and snowball forwarding); this almost certainly over-represents individuals with above-average digital engagement and limits generalizability. Fourth, complete-case analysis with 17.3% exclusion (54 of 312 respondents) may introduce bias if missingness is systematic, even though included and excluded participants did not differ on age or gender distribution. Fifth, no IP-based de-duplication, attention-check items, minimum-time thresholds, or straight-line response detection were implemented; inattentive responding or duplicate participation cannot be completely ruled out. 

Sixth, and most importantly for the substantive conclusions of this work, several theoretically central psychological covariates were not measured: perfectionism, obsessive–compulsive symptoms, dietary restraint, body dissatisfaction, drive for thinness, drive for muscularity, health anxiety, weight stigma, current dieting behavior, supplement use, and prior eating-disorder history. Because these constructs are theoretically and empirically linked to both addictive-like social media use and orthorexia tendency, their absence means that the regression models reported here provide only a partial test of the social media–orthorexia relationship and may underestimate the true associations of interest. Seventh, the BSMAS captures generalized addictive-like social media engagement; the present study did not measure content-specific or platform-specific exposure (e.g., fitspiration, clean-eating communities, influencer nutrition content, body-transformation imagery, fitness-related video feeds), which the literature suggests may be the more proximal correlates of orthorexia tendency. Eighth, some subgroups were small (notably obesity, *n* = 16, and postgraduate, *n* = 6); precision for these post hoc contrasts is correspondingly limited. Finally, multiple bivariate tests increase the risk of false-positive findings, and the small subscale correlations should be interpreted with appropriate caution. 

Future studies should combine BSMAS-type generalized addiction-symptom indices with validated indicators of exposure to nutrition/fitness content (platform type, frequency of engagement with “clean eating” content, follow patterns for influencer accounts, body-comparison behaviors), and incorporate the psychological covariates listed above. Longitudinal and experience-sampling designs would help clarify temporal ordering, and measuring gender beyond a binary item would improve inclusivity. Moderation by gender, BMI, and content exposure across ONI subscales is also a priority for future work. 

## 5. Conclusions

In this cross-sectional study of Turkish adults, orthorexia-related symptoms were associated with gender and weight status. In contrast, addictive-like social media use did not differ by gender or BMI category. Men reported higher ONI total scores and higher BMI than women, and the ONI total was higher in the overweight and obesity groups than in the underweight group. Although BMI showed a small positive bivariate association with ONI total, the BSMAS index of addictive-like social media use was not independently associated with overall orthorexia tendency after adjustment for BMI and lifestyle covariates. The present data support a restrained conclusion: in this sample, a generalized measure of addictive-like social media use was not independently associated with overall orthorexia tendency. Whether platform-specific and content-specific social media variables explain more variance in orthorexia-related outcomes and whether psychological constructs such as perfectionism, dietary restraint, and body dissatisfaction operate as moderators or third-variable confounders should be tested directly in future studies. 

## Figures and Tables

**Table 1:** Participant characteristics (*n* = 258).

Variable	Value
**Continuous variables**	
**Age (years)**	23.96 ± 9.36
**Height (m)**	1.71 ± 0.11
**Weight (kg)**	69.20 ± 16.60
**BMI (kg·m^−2^)**	23.46 ± 4.09
**BSMAS total (6–30)**	18.03 ± 6.20
**ONI total (24–96)**	47.74 ± 13.75
**ONI Behaviors (9–36)**	19.06 ± 5.36
**ONI Impairments (10–40)**	18.20 ± 6.28
**ONI Emotions (5–20)**	10.48 ± 3.58
**Categorical variables**	
**Gender**	
Women	139 (53.9%)
Men	119 (46.1%)
**Education**	
Primary/lower	27 (10.5%)
High school	166 (64.3%)
University	59 (22.9%)
Postgraduate	6 (2.3%)
**Physical activity frequency**	
None	31 (12.0%)
1–2 days/week	80 (31.0%)
3–4 days/week	75 (29.1%)
5–6 days/week	44 (17.1%)
Daily	28 (10.9%)
**Smoking status**	
Never	147 (57.0%)
Former	32 (12.4%)
Current	79 (30.6%)
**Alcohol use**	
None	176 (68.2%)
Occasional	66 (25.6%)
Weekly+	16 (6.2%)
**BMI**	
Underweight (<18.5)	24 (9.3%)
Normal weight (18.5–24.9)	157 (60.9%)
Overweight (25.0–29.9)	61 (23.6%)
Obesity (≥30.0)	16 (6.2%)

**Table 2:** Internal consistency (Cronbach’s α).

Scale	Items	Cronbach’s α
BSMAS	6	0.872
ONI total	24	0.946
ONI Behaviors	9	0.880
ONI Impairments	10	0.909
ONI Emotions	5	0.833

Note. α values ≥ 0.70 are commonly considered acceptable for group-level comparisons.

**Table 3:** Gender differences in study outcomes (Welch’s *t*-test).

Outcome	Men (*n* = 119) Mean ± SD	Women (*n* = 139) Mean ± SD	Welch t (df)	*p*	Hedges g
BSMAS total	18.20 ± 6.08	17.88 ± 6.32	0.42 (252.5)	0.675	0.052
ONI total	51.03 ± 15.23	44.91 ± 11.68	3.58 (219.2)	<0.001	0.454
ONI Behaviors	20.39 ± 5.81	17.92 ± 4.67	3.71 (225.6)	<0.001	0.470
ONI Impairments	19.66 ± 6.96	16.94 ± 5.35	3.48 (219.5)	<0.001	0.442
ONI Emotions	10.98 ± 3.75	10.05 ± 3.38	2.08 (240.0)	0.038	0.261
BMI (kg·m^−2^)	24.97 ± 3.78	22.17 ± 3.92	5.82 (252.3)	<0.001	0.723

Note. Hedges g > 0 indicates higher values in men. Mean ± SD is shown.

**Table 4:** Omnibus group comparisons (Welch’s ANOVA; Kruskal–Wallis as a sensitivity check) for BSMAS total, ONI total, and BMI as outcomes across gender, BMI category (BSMAS and ONI total only), education, physical activity frequency, smoking status, and alcohol use.

Factor	Outcome	Test	Statistic	df1	df2	*p* (Welch)	H (K–W)	*p* (K–W)	ε^2^ (K–W)
Gender	BSMAS total	Welch’s ANOVA	0.18	1	252.5	0.675	0.66	0.416	−0.00
	ONI total	Welch’s ANOVA	12.78	1	219.2	<0.001	10.03	0.002	0.04
	BMI	Welch’s ANOVA	33.89	1	252.3	<0.001	37.94	<0.001	0.14
BMI	BSMAS total	Welch’s ANOVA	0.12	3	48.9	0.948	0.13	0.989	−0.01
	ONI total	Welch’s ANOVA	4.82	3	49.2	0.005	15.31	0.002	0.05
Education	BSMAS total	Welch’s ANOVA	0.58	3	21.0	0.634	2.07	0.558	−0.00
	ONI total	Welch’s ANOVA	0.38	3	22.6	0.768	2.13	0.546	−0.00
	BMI	Welch’s ANOVA	8.01	3	22.6	<0.001	23.57	<0.001	0.08
Physical activity frequency	BSMAS total	Welch’s ANOVA	0.58	4	92.8	0.680	1.69	0.793	−0.01
	ONI total	Welch’s ANOVA	0.80	4	94.3	0.528	3.38	0.497	−0.00
	BMI	Welch’s ANOVA	0.90	4	93.8	0.470	3.72	0.445	−0.00
Smoking status	BSMAS total	Welch’s ANOVA	1.49	2	81.7	0.230	3.52	0.172	0.01
	ONI total	Welch’s ANOVA	0.20	2	80.4	0.819	0.44	0.802	−0.01
	BMI	Welch’s ANOVA	2.50	2	83.7	0.088	5.89	0.053	0.02
Alcohol use	BSMAS total	Welch’s ANOVA	2.72	2	38.8	0.079	5.41	0.067	0.01
	ONI total	Welch’s ANOVA	1.68	2	38.9	0.200	3.10	0.212	0.00
	BMI	Welch’s ANOVA	0.55	2	40.0	0.580	1.92	0.383	−0.00

Note. Welch’s ANOVA was used to account for unequal variances. Kruskal–Wallis (K–W) statistics and ε^2^ are reported as non-parametric sensitivity checks. Under the BMI category as the grouping factor, only BSMAS total and ONI total are reported as outcomes; BMI is not included as an outcome because BMI categories are derived from BMI, making such a comparison tautological by construction.

**Table 5:** ONI total across BMI categories.

BMI	*n*	Mean ± SD	Median
Underweight (<18.5)	24	42.17 ± 12.39	42.0
Normal weight (18.5–24.9)	157	46.58 ± 13.51	47.0
Overweight (25.0–29.9)	61	51.20 ± 14.30	49.0
Obesity (≥30.0)	16	54.25 ± 11.32	55.5

Note. BMI categories: Underweight (<18.5), Normal weight (18.5–24.9), Overweight (25.0–29.9), Obesity (≥30.0).

**Table 6:** BMI across education groups.

Education	*n*	Mean ± SD	Median
Primary/lower	27	21.75 ± 5.00	19.7
High school	166	23.09 ± 3.77	22.6
University	59	24.93 ± 4.12	24.2
Postgraduate	6	26.99 ± 2.35	26.8

Note. Education categories were harmonized as Primary/lower, High school, University, and Postgraduate.

**Table 7:** Holm-adjusted post hoc pairwise comparisons for significant omnibus effects.

Factor	Outcome	Group 1	Group 2	Welch t	Hedges g	*p* (Raw)	*p* (Holm)
BMI	ONI total	Underweight (<18.5)	Normal weight (18.5–24.9)	−1.60	−0.329	0.118	0.237
		Underweight (<18.5)	Overweight (25.0–29.9)	−2.89	−0.648	0.006	0.029
		Underweight (<18.5)	Obesity (≥30.0)	−3.18	−0.988	0.003	0.019
		Normal weight (18.5–24.9)	Overweight (25.0–29.9)	−2.17	−0.335	0.032	0.096
		Normal weight (18.5–24.9)	Obesity (≥30.0)	−2.53	−0.573	0.020	0.080
		Overweight (25.0–29.9)	Obesity (≥30.0)	−0.91	−0.220	0.373	0.373
Education	BMI	Primary/lower	High school	−1.33	−0.335	0.195	0.195
		Primary/lower	University	−2.89	−0.715	0.006	0.024
		Primary/lower	Postgraduate	−3.85	−1.092	0.001	0.008
		High school	University	−3.03	−0.477	0.003	0.016
		High school	Postgraduate	−3.89	−1.041	0.008	0.024
		University	Postgraduate	−1.87	−0.506	0.096	0.193

Note. *p* (Holm) is the multiplicity-adjusted *p*-value. Hedges’ g effect-size magnitudes follow Cohen (1988). Pairwise contrasts are reported only for the two significant omnibus effects (BMI category on ONI total; education on BMI). BMI is not contrasted across BMI categories because the grouping factor is itself defined from BMI.

**Table 8:** Pearson correlations among key study variables.

Variable 1	Variable 2	Pearson r	95% CI	*p*
BSMAS total	ONI total	0.092	[−0.031, 0.211]	0.143
	ONI Behaviors	−0.027	[−0.149, 0.095]	0.661
	ONI Impairments	0.139	[0.017, 0.257]	0.025
	ONI Emotions	0.148	[0.027, 0.266]	0.017
BMI	ONI total	0.136	[0.014, 0.254]	0.029
Age	BSMAS total	−0.172	[−0.288, −0.051]	0.006
	ONI total	0.022	[−0.100, 0.144]	0.725
	BMI	0.392	[0.284, 0.491]	<0.001

Note. CI = confidence interval. Correlation magnitude follows Cohen (1988): r ≈ 0.10 = small, 0.30 = medium, 0.50 = large.

**Table 9:** Multiple regression predicting ONI total (HC3 robust standard errors).

Predictor	B	SE (HC3)	95% CI Low	95% CI High	*p*	VIF
Intercept	35.863	6.413	23.293	48.433	<0.001	−
Gender (Men vs. Women)	7.142	2.218	2.795	11.489	0.001	1.42
Physical activity: 1–2 days/week	−2.030	2.987	−7.884	3.825	0.497	2.21
Physical activity: 3–4 days/week	0.365	3.248	−6.000	6.731	0.910	2.18
Physical activity: 5–6 days/week	0.897	3.960	−6.864	8.658	0.821	1.72
Physical activity: Daily	−1.323	3.831	−8.831	6.185	0.730	1.59
Smoking: Former	0.183	2.990	−5.677	6.042	0.951	1.13
Smoking: Current	−1.321	2.210	−5.652	3.010	0.550	1.21
Alcohol use: Occasional	−4.958	2.247	−9.362	−0.554	0.027	1.27
Alcohol use: Weekly+	−5.043	4.605	−14.069	3.982	0.273	1.18
Education: Primary/lower	2.926	2.686	−2.340	8.191	0.276	1.31
Education: University	−2.106	2.743	−7.483	3.270	0.443	1.45
Education: Postgraduate	−6.630	7.185	−20.713	7.453	0.356	1.09
BSMAS total	0.246	0.167	−0.082	0.574	0.141	1.10
BMI (kg·m^−2^)	0.244	0.232	−0.211	0.699	0.293	1.61
Age (years)	0.051	0.119	−0.183	0.285	0.669	1.55

Note. Model fit: R^2^ = 0.114, adjusted R^2^ = 0.059. VIF column added on revision; all VIFs < 2.5. No influential cases were detected (Cook’s D and standardized dfBETAs within thresholds).

**Table 10:** Multiple regression predicting BSMAS total (HC3 robust standard errors).

Predictor	B	SE (HC3)	95% CI Low	95% CI High	*p*	VIF
Intercept	22.069	2.763	16.653	27.485	<0.001	−
Gender (Men vs. Women)	0.603	0.994	−1.345	2.551	0.544	1.42
Physical activity: 1–2 days/week	−1.567	1.472	−4.453	1.319	0.287	2.21
Physical activity: 3–4 days/week	−1.270	1.526	−4.261	1.720	0.405	2.18
Physical activity: 5–6 days/week	−1.978	1.926	−5.753	1.797	0.304	1.72
Physical activity: Daily	−2.453	2.014	−6.401	1.494	0.223	1.59
Smoking: Former	1.250	1.301	−1.301	3.800	0.337	1.13
Smoking: Current	0.571	1.014	−1.416	2.558	0.574	1.21
Alcohol use: Occasional	0.897	1.066	−1.194	2.987	0.400	1.27
Alcohol use: Weekly+	3.170	1.850	−0.457	6.796	0.087	1.18
Education: Primary/lower	−0.001	1.351	−2.648	2.646	0.999	1.31
Education: University	1.529	1.373	−1.162	4.220	0.265	1.45
Education: Postgraduate	−0.545	4.742	−9.839	8.749	0.909	1.09
BMI (kg·m^−2^)	0.008	0.102	−0.192	0.209	0.935	1.61
Age (years)	−0.173	0.060	−0.291	−0.055	0.004	1.55

Note. Model fit: R^2^ = 0.079, adjusted R^2^ = 0.025.

**Table 11:** Multiple regression predicting BMI (HC3 robust standard errors).

Predictor	B	SE (HC3)	95% CI Low	95% CI High	*p*	VIF
Intercept	18.786	1.196	16.442	21.130	<0.001	−
Gender (Men vs. Women)	2.141	0.562	1.040	3.242	<0.001	1.42
Physical activity: 1–2 days/week	−0.281	0.806	−1.861	1.300	0.728	2.21
Physical activity: 3–4 days/week	0.087	0.861	−1.599	1.774	0.919	2.18
Physical activity: 5–6 days/week	0.529	1.050	−1.530	2.588	0.615	1.72
Physical activity: Daily	−0.635	0.968	−2.533	1.263	0.512	1.59
Smoking: Former	0.279	0.732	−1.157	1.714	0.703	1.13
Smoking: Current	0.426	0.619	−0.787	1.640	0.491	1.21
Alcohol use: Occasional	−0.807	0.542	−1.870	0.256	0.137	1.27
Alcohol use: Weekly+	0.058	1.352	−2.593	2.708	0.966	1.18
Education: Primary/lower	−0.799	0.883	−2.530	0.933	0.366	1.31
Education: University	−0.649	0.857	−2.329	1.030	0.449	1.45
Education: Postgraduate	−1.043	1.742	−4.457	2.371	0.549	1.09
Age (years)	0.168	0.049	0.072	0.264	<0.001	1.55

Note. Model fit: R^2^ = 0.246, adjusted R^2^ = 0.206.

## Data Availability

The data presented in this study are available upon request from the corresponding author due to confidentiality concerns.

## Abbreviations

The following abbreviations are used in this manuscript:

BMI	Body Mass Index
BSMAS	Bergen Social Media Addiction Scale
ONI	Orthorexia Nervosa Inventory
TurkStat	Turkish Statistical Institute
WHO	World Health Organization
